# Neuroendoscopic Trans-ventricular Cyst Drainage Prior to Tumour Resection Using a Two-Staged Approach for Cystic Craniopharyngioma: A Case Report and Literature Review

**DOI:** 10.7759/cureus.102863

**Published:** 2026-02-02

**Authors:** Saeed Javid, Dace Dimante, Soha Zahid, Ahmed Eweiss, Alireza Shoakazemi

**Affiliations:** 1 Trauma and Orthopaedics, St Peter's Hospital, Surrey, GBR; 2 Neurosurgery, Queen’s Hospital, Barking, Havering and Romford University Hospitals Trust, London, GBR; 3 Otorhinolaryngology, Queen’s Hospital, Barking, Havering and Romford University Hospitals Trust, London, GBR

**Keywords:** adamantinomatous, craniopharyngioma, cystic craniopharyngioma, endoscopic, neuroendoscopy, transsphenoidal resection

## Abstract

Craniopharyngiomas are benign suprasellar tumours that can present with symptoms of raised intracranial pressure, visual impairment, or endocrine dysfunction. They are classified into the more common adamantinomatous and less common papillary tumours. Cystic variants may cause obstructive hydrocephalus, necessitating urgent intervention. This video case-based review presents the case of a 61-year-old male who presented with a two-month history of headaches, cognitive decline, and visual changes. Imaging revealed a large 2.8 x 2.2 x 2.2 cm suprasellar mass with cystic components extending into the third ventricle - consistent with a craniopharyngioma, causing bilateral foraminal obstruction and acute hydrocephalus. An urgent right frontal endoscopic septum pellucidostomy, cyst drainage, and ventriculoperitoneal shunt insertion achieved rapid symptomatic relief. Histopathology confirmed a World Health Organization* (*WHO) Grade 1 adamantinomatous craniopharyngioma. Following neurological stabilisation, a planned second-stage extended transsphenoidal resection achieved near-total removal. Postoperative radiotherapy was delivered to the tumour residuum. Endocrine sequelae, including hypopituitarism and diabetes insipidus, were managed with hormone replacement. At one-year follow-up, the patient remained radiologically stable with no recurrence. A review of current literature further supports neuroendoscopic transventricular cyst aspiration as a temporising measure in cyst-dominant craniopharyngiomas, particularly in the setting of acute hydrocephalus. This strategy offers rapid decompression, symptom relief, facilitating safer, elective definitive surgery. However, as a sole intervention, it is rarely curative, and recurrence risk remains high without subsequent resection and/or radiotherapy. A planned, two-stage approach combining neuroendoscopic decompression with definitive tumour control represents a safe and effective management strategy for giant cystic craniopharyngiomas.

## Introduction

Craniopharyngiomas are benign, dysontogenetic brain tumours that occur in both adults and children alike. They represent up to 10% of all childhood brain tumours, and up to 3% of adult brain tumours [1-3]. They can take two to three years or even longer to manifest themselves before a definitive diagnosis is ultimately made [2]. They are exceedingly rare even within the realm of neurosurgery, with approximately 0.5 to two new cases per million population each year [4, 5].

There are two histological variants, namely adamantinomatous craniopharyngiomas (ACP), and papillary craniopharyngiomas (PCP). The adamantinomatous variant is much more common in children, showing cystic foci, calcifications, and “wet keratin”. On the contrary, the papillary subtype is much more common in adults - almost exclusively - and is typically more solid and less likely to calcify [1]. 

Despite their indolent histology and slow-growing nature, craniopharyngiomas can cause significant neurological and endocrine sequelae due to their critical suprasellar location. The adamantinomatous subtype in particular, frequently demonstrates prominent cystic components, which may expand rapidly and compress adjacent structures such as the optic chiasm, hypothalamus, and third ventricle. These can present with mass-effect symptoms such as headaches, bitemporal hemianopsia, or endocrine abnormalities. In turn, this can contribute to significant morbidity for patients with craniopharyngiomas, despite their overall favourable prognosis and long-term survival. In some cases, cyst enlargement results in acute obstructive hydrocephalus, leading to raised intracranial pressure, visual decline, and cognitive deterioration. These presentations often require urgent intervention to restore cerebrospinal fluid flow and stabilise the patient before definitive tumour treatment can be undertaken. In this context, novel techniques such as transventricular neuroendoscopic drainage have emerged as valuable minimally invasive options for acute cyst decompression [1, 2].

Surgical resection has traditionally represented the mainstay of long-term management for craniopharyngiomas, with or without adjuvant radiotherapy depending on individual patient factors and tumour characteristics. However, immediate radical excision may not be feasible or safe in clinically unstable patients or in those with significant cyst-related mass effect. This case-based review contributes to the growing evidence supporting novel, two-staged planned treatment strategies for cyst-dominant craniopharyngiomas. It highlights the value of urgent neuroendoscopic transventricular cyst drainage as a minimally invasive stabilising intervention to achieve rapid decompression and relieve acute obstructive hydrocephalus [2].

Furthermore, it illustrates how an innovative two-staged approach allows neurological recovery and safe interval planning for definitive resection, thereby reducing perioperative risk compared with immediate radical excision in unstable patients. In addition, coordinated multidisciplinary care - particularly endocrinology and ophthalmology involvement - remains essential to optimise management of associated visual and pituitary dysfunction [3].

## Case presentation

**Figure 1:**
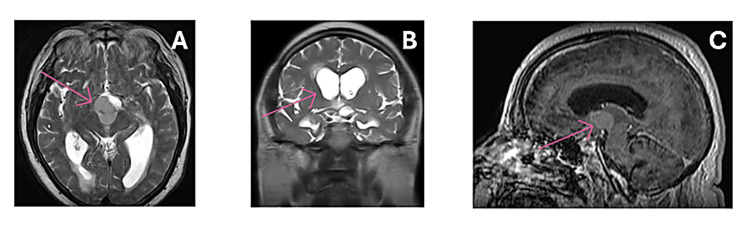
Preoperative MRI scans, prior to initial transventricular endoscopic cyst drainage. A: shows the acute hydrocephalus on an MRI (axial slice) performed on admission. The red arrow points to the suprasellar lesion. B: acute obstructive hydrocephalus (ventricular dilatation) shown on the coronal MRI slice. This was secondary to the large cystic craniopharyngioma lesion pictured in image C. C: shows the large suprasellar mass extending into the third ventricle on a sagittal MRI slice, causing the secondary acute obstructive hydrocephalus.

We present the case of a 61-year-old gentleman who presented to the emergency department with a subacute history of morning headaches, which had significantly worsened on the day of admission, and memory issues. Despite not reporting any overt eyesight problems, his family had noticed that his driving ability had worsened over the past few weeks. Furthermore, he was oriented to person and place, but not to date or time. Due to being obtunded on admission, an emergency MRI was performed, which revealed a large suprasellar mass extending into the third ventricle. This measured at 2.8 x 2.2 x 2.2 cm, with appearances initially suggestive of a large colloid cyst (Figure 1), which had consequently led to a secondary acute obstructive hydrocephalus. After further characterisation, the appearances were instead deemed to be consistent with those of a craniopharyngioma.

Due to the acuity of the patient’s clinical presentation and symptoms, an urgent right frontal endoscopic septum pellucidostomy was performed on the same day of presentation. A subsequent transventricular cyst drainage using a paediatric nasogastric tube down the endoscope, and a right frontal ventriculoperitoneal shunt with medium-high fixed Codman Hakim valve was inserted for cerebrospinal fluid (CSF) diversion. All postoperative scans and investigations were satisfactory, and he was discharged for clinic follow-up in the subsequent weeks, with a repeat MRI. In addition, the patient was also discussed at the regional neuro-oncology multi-disciplinary team meeting (MDT), once histology samples confirmed a central nervous system (CNS) World Health Organization (WHO) Grade 1 adamantinomatous craniopharyngioma, and a management plan was implemented.

**Figure 2:**
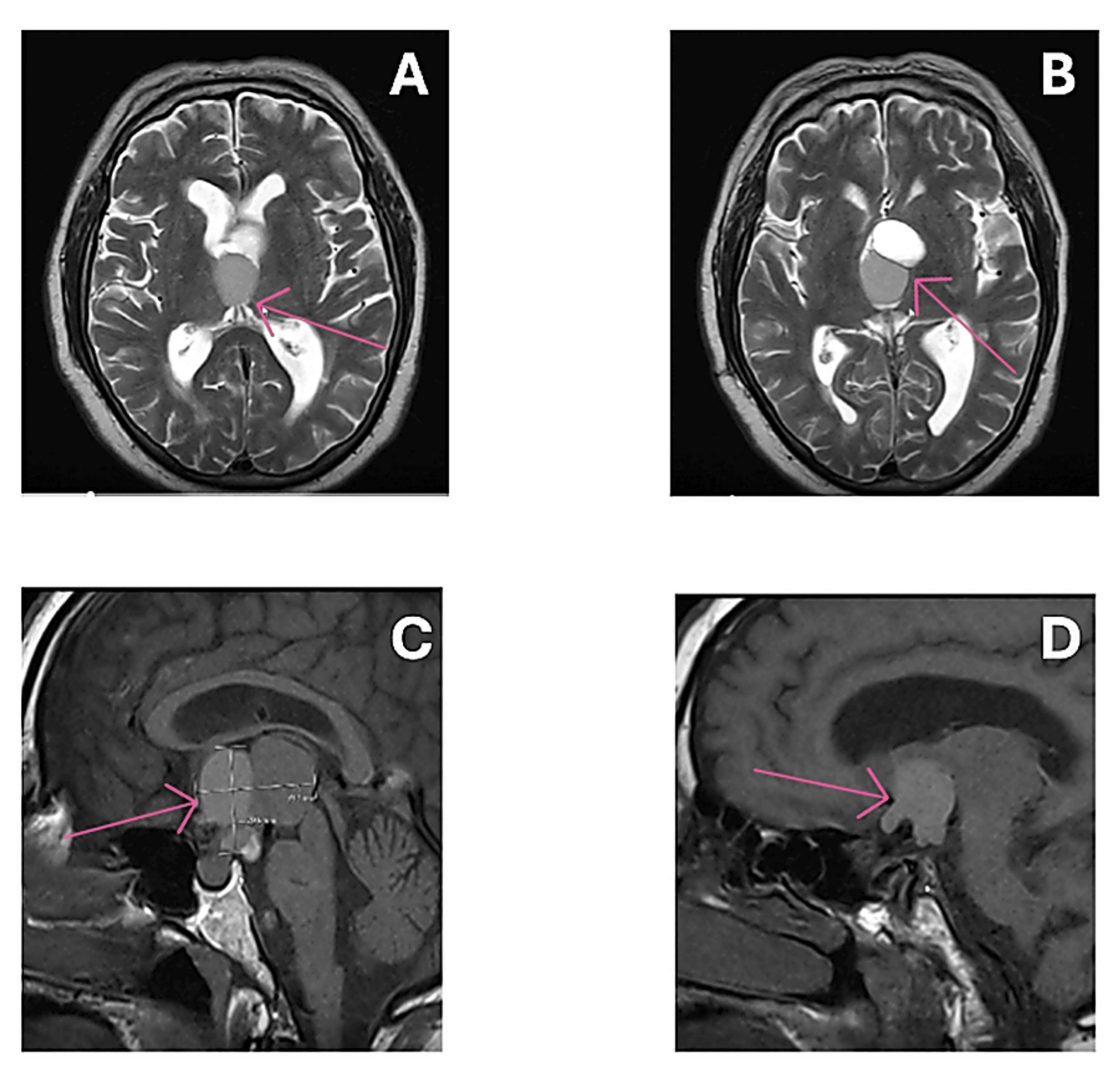
MRI scans prior to the second operation (trans-sphenoidal tumour resection), showing some progression of the disease, with an increase in the size of the craniopharyngioma. A and B: show the presence of the cystic craniopharyngioma in the suprasellar region, depicting the tumour on an axial slice. C and D: further characterise the craniopharyngioma tumour on a sagittal MRI slice. At this point, the patient's symptoms had been progressing slightly, warranting a definitive surgical resection, following the initial transventricular aspiration of the craniopharyngioma cyst a few months before.

At the four-week clinic follow-up, the patient remained clinically well but did report non-specific intermittent frontal headaches, which were responsive to simple oral analgesics, as well as a general decline in vision. An interval CT head before clinic demonstrated improved ventricular size with no acute pathology. He was also scheduled for an ophthalmology review, and on examination had “good stable visual acuity in the right eye but slightly reduced in the left eye” with right eye 6/9 (6/6 pinhole) and left eye 6/18 (6/9) pinhole. Further investigations in the following weeks included a neuro-ophthalmology review, an MRI (Figure 2) with interval pituitary bloods, and a neurosurgical follow-up to assess whether the craniopharyngioma required any further treatment.

Though the patient remained stable, he continued to have visual symptoms, which led him to present to the emergency department over the course of a few weeks to months. The symptoms he presented with were a two-day history of worsening dizziness, nausea, and headaches, responding to simple analgesics, and no concerning features. He was discharged with advice, and a CT that day showed no acute intracranial findings, with overall improvement in the ventricular dilatation, as well as resolution of the intraventricular haematoma noticed postoperatively.

This followed a trend, with the patient noticing a gradual deterioration of his vision, particularly in the left eye. Therefore, a few months after his initial presentation to hospital where he had a surgical endoscopic cyst drainage and decompression, the patient had opted for definitive craniopharyngioma surgery. This would be in the form of a transsphenoidal resection with insertion of a lumbar drain. The definitive surgical resection was scheduled to be completed earlier, but was delayed due to patient-related factors and circumstances.

**Figure 3:**
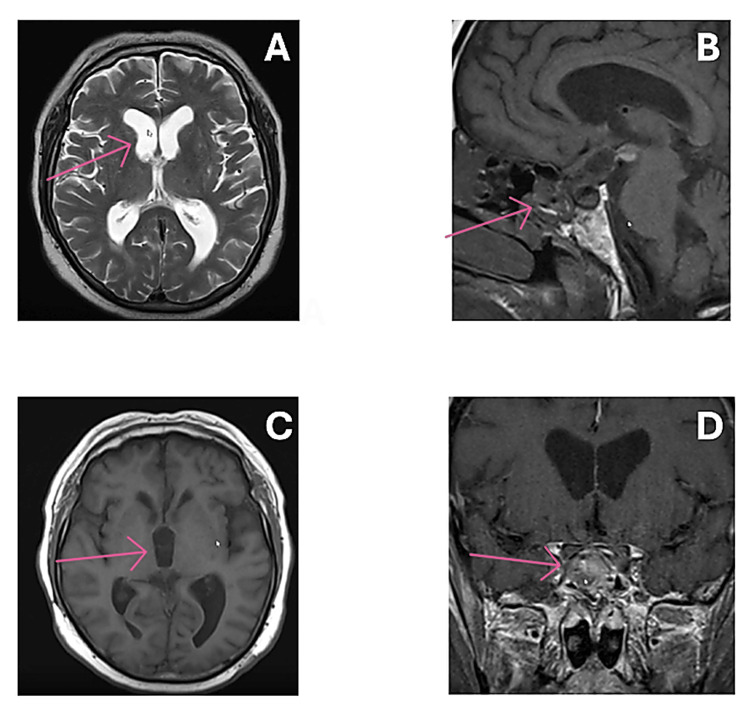
Postoperative MRI scans, approximately 1 month after the second surgical procedure (trans-sphenoidal resection of the craniopharyngioma). Scans show resolution of the cystic component of the craniopharyngioma. A and C: show overall resolution of ventricular dilatation, with normalisation of the size of the ventricles, since definitive surgical resection of the tumour (axial slice). B: shows the resolution of the cystic component of the craniopharyngioma in the sagittal plane, in addition to the removal of the suprasellar mass (craniopharyngioma tumour). D: shows the latest MRI scans (coronal plane), following the surgical resection of the suprasellar mass (craniopharyngioma).

The patient was admitted in early December 2023 for a planned endoscopic extended transsphenoidal resection of a craniopharyngioma. A near-total excision was achieved, with a small residual component noted in the region of the hypothalamus and brainstem. An MRI performed just before definitive surgical craniopharyngioma resection is shown below (Figure 3).

Immediate postoperative CT revealed dilatation of the lateral ventricles with evidence of intraventricular haemorrhage. The previously identified cystic lesion within the suprasellar cistern, noted on MRI dated 03/12/2023, demonstrated interval high-density changes consistent with haemorrhagic transformation. Extension into the floor of the third ventricle was also observed. Despite these radiological findings, the patient remained neurologically intact with a Glasgow Coma Scale (GCS) score of 15 and stable visual function. There were no clinical signs of CSF leakage. In view of the stable clinical status, a conservative approach was adopted, and a repeat CT head was performed the following day.

Subsequent imaging did not demonstrate any progression warranting further surgical intervention, and the neurosurgical team concluded that haematoma evacuation would not provide clinical benefit. The patient continued to recover in the intensive care unit and was eventually discharged with plans for outpatient follow-up, including an endocrine review. He commenced desmopressin therapy during his recovery for hormone replacement, due to postoperative pituitary failure, where he developed central diabetes insipidus.

The patient was subsequently re-discussed at the multidisciplinary team meeting. The outcome of the MDT was that, in view of the rapid regrowth of the lesion and the presence of residual disease displayed on the most recent MRI, adjuvant radiotherapy would be recommended. A course of postoperative radiotherapy was planned and received, delivering 50 Gy in 30 fractions. He was counselled that, without radiotherapy, the lesion was likely to recur. The potential risks of radiotherapy were also discussed, including the possibility of further pituitary insufficiency necessitating additional hormone replacement beyond his current hydrocortisone and desmopressin therapy.

**Figure 4:**
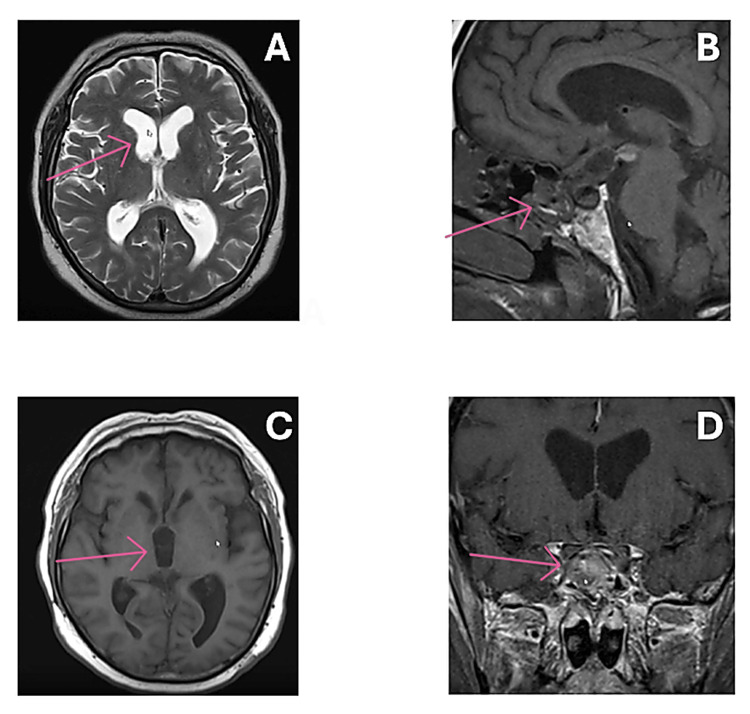
Postoperative MRI scans, approximately 1 month after the second surgical procedure (trans-sphenoidal resection of the craniopharyngioma). Scans show resolution of the cystic component of the craniopharyngioma. Images A and C show the overall resolution of ventricular dilatation, with normalisation of the size of the ventricles, since definitive surgical resection of the tumour. Image B show resolution of the cystic component of the craniopharyngioma in the sagittal plane, in addition to the removal of the suprasellar mass (craniopharyngioma tumour). Image D show the latest MRI scans (coronal plane), following the surgical resection of the suprasellar mass (craniopharyngioma).

He tolerated adjuvant radiotherapy well. Interval MRI demonstrated a reduction in the volume of the residual enhancing tissue, with resolution of the cystic component (Figure 4). Clinically, he reported no recurrence of headaches, which had been his presenting symptom on admission. At the endocrine follow-up, just over one year following definitive tumour debulking, the patient was found to have developed hypopituitarism (a natural sequelae of this type of surgical procedure), and was therefore commenced on full hormone replacement therapy. He remained stable and well.

## Discussion

Surgical strategies form the mainstay of craniopharyngioma management, oftentimes combined with additional adjuvant radiotherapy to further reduce tumour size postoperatively [2]. However, evolving neuro-endoscopic techniques have come to the fore in recent years, as alternative or adjunctive treatment strategies in these complex patients [6, 7].

We conducted a targeted review of the current literature, detailing the role of transventricular endoscopic aspiration of cystic craniopharyngioma before further surgical management, evaluating the benefits and disadvantages of such an approach. The literature search was conducted between April and May 2025, and studies were identified through a comprehensive search of four electronic databases: MEDLINE (via PubMed), Scopus, Web of Science, and the Cochrane Library.

The search combined MeSH terms and free-text keywords. Search terms included combinations of “craniopharyngioma”, “cystic”, “neuroendoscopy”, “transventricular”, “endoscopic”, “adamantinomatous”, “hydrocephalus”, “drainage, or “decompression”. Both paediatric and adult populations were included. Additional sources were identified by manually screening the reference lists of selected articles. Eligible studies comprised case reports, case series, and narrative or systematic reviews describing neuroendoscopic aspiration in the management of cystic craniopharyngioma. Sixteen articles meeting these criteria were included and analysed in the literature review [6].

The overall rationale for neuro-endoscopic intraventricular aspiration of craniopharyngioma cyst before definitive surgical management is that it allows rapid decompression of cystic components, which may be causing an obstructive hydrocephalus at presentation [7]. Furthermore, it can facilitate a staged, elective surgical craniopharyngioma resection later, with interim preoperative stabilisation and optimisation of the patient [7-9].

One of the major benefits of initially performing transventricular aspiration before definitive surgery is that it can allow immediate symptomatic relief from a large craniopharyngioma cyst causing acute obstructive hydrocephalus. In addition to decompression of the cyst and hydrocephalus, studies have shown significant benefit from this approach in patients presenting with visual issues on admission. Jugović et al. demonstrated how early transventricular decompression of a large cystic craniopharyngioma leads to favourable outcomes. In this study, a 56-year-old with bilateral complete blindness for three days, on a background of enlarging cystic craniopharyngioma, underwent an emergency decompression of her optical apparatus. Postoperatively, her visual acuity significantly improved in one of her eyes [7]. This study described what was an effective improvement in visual impairment post-transventricular endoscopic aspiration and established that vision loss can still be recovered within a 72-hour window.

A case-based review by Noureldine et al. highlighted the impact of endoscopic transventricular cyst aspiration in the paediatric population, reporting no complications over a two-year follow-up period for a nine-year-old female with a large cystic craniopharyngioma, who was managed using a transventricular approach. A review of the literature in the study also showed an overall reduced recurrence and complication rate in this neuroendoscopic transventricular approach for cystic-dominant craniopharyngiomas, when compared to other surgical modalities, including Ommaya reservoir insertion and microsurgery [8].

Delitala et al. described seven patients undergoing a transventricular endoscopic aspiration as a single-staged intervention in the management of patients presenting with craniopharyngioma. The authors noted that endoscopic decompression allowed for the creation of space for safer manipulation and planning of further surgical procedures. In one of the cases described, the cyst reduction had successfully facilitated a subsequent craniotomy [10]. Thus, the authors advocate for the use of a neuroendoscopic transventricular approach as a minimally invasive first-line step, especially in cyst-dominant tumours.

Transventricular cyst drainage is particularly advantageous in paediatric craniopharyngiomas, which account for up to 10% of childhood brain tumours and frequently present with hydrocephalus [2]. Neuroendoscopic aspiration offers a safe initial intervention in fragile children with raised intracranial pressure, providing rapid decompression and neurological stabilisation. A paediatric-only series reports hydrocephalus on admission in 17.1% to up to 75% of cases [11, 12]. Gangemi et al. illustrated this in a six-year-old with a giant cystic craniopharyngioma, where initial endoscopic transventricular drainage relieved intracranial pressure, improved CSF circulation, reduced tumour volume, and decreased tension on critical neuroanatomy, thereby enabling a safer second-stage microsurgical resection [13]. The authors concluded that proceeding directly to radical resection would have carried higher morbidity, highlighting neuroendoscopy as an effective temporising strategy in high-risk paediatric patients before definitive surgery and/or radiotherapy.

Further support for neuroendoscopy in paediatric craniopharyngiomas arises from a paradigm shift away from radical single-stage resections toward more conservative, minimally invasive strategies. Paediatric tumours are commonly of the adamantinomatous subtype, characterised by cystic, calcified, and infiltrative growth with close adherence to the hypothalamus, optic apparatus, and pituitary stalk [14]. Consequently, children face a higher risk of hypothalamic injury and endocrine morbidity, including panhypopituitarism and diabetes insipidus, following radical resection. These complications are particularly detrimental in children, leading to severe obesity, cognitive and behavioural impairment, and reduced long-term quality of life [15]. As radical resections are associated with higher neuroendocrine morbidity in children than adults, this supports the use of transventricular neuroendoscopy within a staged or combined treatment strategy to maximise disease control while minimising morbidity in the paediatric population.

Despite this, there are some limitations to performing neuroendoscopic transventricular aspiration before definitive surgical management of a craniopharyngioma. Bianchi et al. described the use of multiple treatment modalities, including intracystic therapies, surgery, and endoscopic drainage. It reported that cystic decompression is rarely curative if used alone; and that these cysts can frequently recur if not followed by definitive treatment such as surgical resection or radiotherapy [16]. This may lead to multiple aspirations or revisions, as described by Kulkarni et al., where out of a total of seven patients, cyst recurrence occurred in two, requiring re-aspiration of the cyst via an Ommaya reservoir. Thus, the authors note that cyst re-accumulation may warrant multiple interventions in the absence of definitive treatment [17]. Both studies ultimately show that temporary control can be achieved by utilising transventricular endoscopic drainage, but recurrence is common unless followed by definitive therapy.

Two-staged combined strategy: transventricular cyst aspiration, followed by definitive surgical resection, and/or radiotherapy

With the benefits and limitations of the role of transventricular aspiration in cystic craniopharyngiomas either as a lone intervention or adjunct with surgical resection discussed above, we suggest that a combined strategy- as demonstrated in our case report, and as described by Gangemi et al., and Deopujari et al. is the optimal approach when managing giant craniopharyngiomas with a cystic component causing obstructive hydrocephalus [13, 18, 19].

This combined two-staged approach for cystic-predominant craniopharyngioma is supported by Gangemi et al. and Deopujari et al. This is initially with a transventricular endoscopic decompression (± Ommaya) in the first instance, followed by surgical resection and/or adjuvant radiotherapy for definitive tumour control [13, 19].

In addition to the article by Gangemi et al. as described above, Deopujari et al. reported a five-patient case series of predominantly cystic suprasellar craniopharyngiomas managed using a staged approach. Initial endoscopic cyst aspiration and fenestration, often with Ommaya reservoir placement, provided rapid symptomatic relief and neurological stabilisation. Definitive management with microsurgical resection or adjuvant radiotherapy was then undertaken based on residual disease and patient factors. Cyst decompression reduced tumour size, facilitating safer subsequent intervention and radiotherapy targeting [19]. The authors concluded that this combined two-stage strategy achieved effective cyst control while balancing urgent decompression with durable long-term management.

Transventricular aspiration of cystic craniopharyngioma: current gaps in the literature

Whilst current evidence suggests that neuroendoscopic techniques are an increasingly used and relied-upon option in managing conditions such as cystic craniopharyngioma, there are certain gaps in the current literature that encumber for more conclusive decisions from being made, with respect to the optimal role and timing of intraventricular endoscopic aspiration [10, 17, 18].

Most of the current evidence is primarily based on a case series or retrospective observational studies [10, 16-19]. Consequently, there is an absence of high-level evidence that can determine causality, as well as make unbiased, controlled comparisons of recurrence rates, outcomes, and complication rates between different management strategies. Therefore, there is a necessity for randomised controlled trials, which would clearly and objectively compare patients who undergo neuroendoscopic transventricular cyst aspiration alone, versus patients who undergo a combined strategy involving neuroendoscopy with subsequent surgery or radiotherapy.

Furthermore, there is much variability shown across studies with respect to the age of patients, location of craniopharyngioma (intraventricular extension vs solely suprasellar), and the absence or presence of hydrocephalus, amongst other variables. These inconsistencies restrict our ability to make substantiated, evidence-based clinical conclusions, and make direct comparisons between different modalities very difficult to appreciate [16, 17]. Therefore, future research should aim to stratify patients by their age group, ventricular size, and tumour characteristics (mixed vs cystic), as the current literature is restricted by the lack of long-term follow-up in studies, the absence of randomised controlled trials, and an overall heterogeneity in the tumour and patient characteristics [16-19].

## Conclusions

In summary, this case illustrates the feasibility of transventricular neuroendoscopic cyst drainage as a temporising measure in an acutely unwell patient with a large cystic craniopharyngioma causing raised intracranial pressure and obstructive hydrocephalus. Transventricular neuroendoscopic decompression was followed by rapid symptomatic improvement and resolution of ventricular dilatation, allowing neurological stabilisation and deferral of definitive treatment to a more controlled, elective setting.

However, residual tumour requiring adjuvant radiotherapy and the subsequent development of progressive anterior pituitary failure highlight that neuroendoscopic drainage is not alone curative, and that durable disease control often requires combined, staged management. Whilst a review of the literature on this subject indicates that evidence remains limited, existing reports and physiological rationale suggest that neuroendoscopic decompression may have a role as part of a planned multi-modal strategy - particularly for cyst-dominant or giant lesions and in higher-risk cohorts - though robust comparative data, and higher levels of scientific research evidence are needed to clarify indications and outcomes.
